# Effect of template removal using plasma treatment on the structure and catalytic performance of MCM-22

**DOI:** 10.1039/c8ra00212f

**Published:** 2018-04-24

**Authors:** Min Hu, Binran Zhao, Dong-Yang Zhao, Mei-Ting Yuan, Huiyong Chen, Qing-Qing Hao, Ming Sun, Long Xu, Xiaoxun Ma

**Affiliations:** School of Chemical Engineering, Northwest University Xi’an Shaanxi 710069 China haoqq@nwu.edu.cn maxym@nwu.edu.cn; Chemical Engineering Research Center of the Ministry of Education for Advanced Use Technology of Shanbei Energy Xi’an Shaanxi 710069 China; Shaanxi Research Center of Engineering Technology for Clean Coal Conversion Xi’an Shaanxi 710069 China

## Abstract

To investigate the effect of template removal methods on the structure, properties and catalytic performance of the MCM-22 zeolite, dielectric-barrier discharge (DBD) plasma treatment and thermal calcination have been comparatively studied for the removal of hexamethyleneimine (HMI) from the two-dimensional layered precursor of MCM-22 (MCM-22(P)). The materials were characterized using FT-IR, TG, XRD, N_2_ adsorption at low temperature, NH_3_-TPD, and ^27^Al and ^29^Si MAS NMR. The results revealed that the seven-membered heterocyclic compound HMI can be effectively removed from the MCM-22 zeolite, and the condensation of silanol groups on the neighboring surface of MWW nanosheets can be induced by DBD treatment. Compared with calcination, DBD treatment could preserve the structure well and decrease the formation of extra-framework aluminum. Consequently, the concentration of acidic sites over MCM-22 treated by DBD (MCM-22(DBD)) is higher than that over calcined MCM-22 (MCM-22(C)). Moreover, MCM-22(DBD) possesses a certain amount of external surface area derived from the intercrystal pores due to the inhibiting effect of the condensation of the silanol groups on the external surface of the MCM-22 crystals. The activity and product selectivity of the Fischer–Tropsch (FT) synthesis was investigated over cobalt supported on the obtained MCM-22 zeolites. Compared with Co/MCM-22(C), Co/MCM-22(DBD) shows a higher catalytic activity in the FT synthesis reaction. Moreover, Co/MCM-22(DBD) can effectively decrease CH_4_ selectivity and increase C_5_–C_20_ liquid fuel selectivity.

## Abstract 2

Template removal from MCM-22 using dielectric-barrier discharge (DBD) plasma could decrease the formation of extra-framework aluminum (EFAl) and increase the concentration of the acidic sites and external surface area of MCM-22.

## Graphical abstract

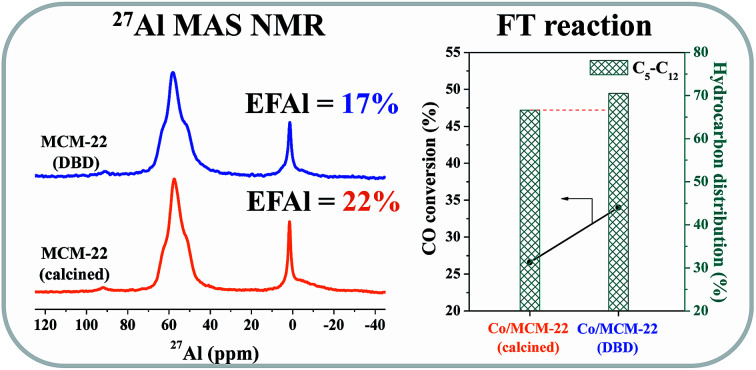

## 1. Introduction

As the first layered zeolite that was discovered, MCM-22 zeolite has received considerable attention in catalysis due to its unique porous structure, which consists of two independent porous systems, both being accessible through 10-membered ring (MR) windows.^1–5^ The as-synthesized precursor of MCM-22, namely MCM-22(P), is a two dimensional (2D) layered structure, which consists of MWW layers kept together by hydrogen bonds between terminal silanol groups (Si–OH) in neighboring surfaces.^1,2^ After removing the structural directing agents (SDAs) located in the interlayer space by thermal calcination, condensation of the silanol groups occurred and the contiguous layers connected, generating the corresponding three-dimensional (3D) MCM-22 zeolite.^3,4^ Generally, thermal calcination is the most widely used method for template removal from zeolites. However, thermal calcination will lead to secondary effects such as the extraction of framework aluminium and partial amorphisation due to the high local temperatures and formation of water in the calcination process.^6,7^ Moreover, another side effect of high temperature calcination is the condensation of silanol groups, which will cause a loss of intercrystal porosity, formation of crystal agglomeration, and a lower total surface area.^6^ Therefore, the above mentioned effects of thermal calcination will influence the structure and acidity of MCM-22, which will further affect its catalytic performance. Corma *et al.*^8,9^ reported that partial dealumination takes place during the calcination of lamellar MCM-22(P). Moreover, the one-pot synthesis of MWW zeolite nanosheets with random arrangements (similar to ITQ-2) has been carried out in recent years.^10,11^ Similarly, 22–34% of the extra-framework Al is found in this zeolite after thermal calcination.^10,11^ Thus, the template removal method for MCM-22 and its derived zeolites requires further optimization to minimize the extent of dealumination.^11^

Several mild methods have been developed for template removal from mesoporous materials, such as liquid extraction using acidic solutions,^12^ sonication,^13^ and the use of supercritical CO_2_.^14,15^ However, these methods are not effective for zeolites due to the small pore size and the stronger interaction between the template and the framework of the zeolite.^6,7^ Different attempts have been made for low-temperature decomposition of the template from zeolites. For instance, in order to reduce the calcination temperature, hydrocracking,^16^ catalytic decomposition,^17^ microwave irradiation,^18^ and the use of oxidative gases such as ozone,^19^ N_2_O, and NO_2_ have been developed for template removal from zeolites.^20–22^ However, these methods still require rather elevated temperatures (>200 °C).

Dielectric-barrier discharge (DBD) plasma is cold plasma, which can be initiated under ambient conditions.^23^ This technique has been extensively studied in catalyst preparation.^24–27^ More importantly, preliminary research confirmed that the template in ZSM-5, beta, and mesoporous MCM-41 can be effectively removed using DBD plasma treatment.^28–31^ Moreover, the highest temperature of the sample during DBD template removal is about 125 °C.^29^ Thus, DBD plasma can effectively decrease the thermal effect on the zeolite during template removal. Generally, the effectivity of template removal can be significantly influenced by the topological structure, and the shape, size and location of the template. It is well known that one kind of template for the synthesis of MCM-22 is hexamethyleneimine (HMI), which is a seven-membered heterocyclic compound. Moreover, the template of HMI is not only located at the sinusoidal channels in the MWW nanosheets, but is also located in the interlayers of the nanosheets of MCM-22(P).^1^ Thus, the interaction between the framework and the template for MCM-22 is different to that for ZSM-5 and beta. Consequently, the effect of template removal using plasma treatment on the structure and catalytic performance of MCM-22 is currently unclear.

Based on this understanding, and in order to investigate the effect of the template removal method on the structure, properties, and catalytic performance of the MCM-22 zeolite, in this work DBD plasma treatment and thermal calcination have been comparatively studied for template removal from the two-dimensional layered MCM-22(P). ^27^Al and ^29^Si MAS NMR has been used to discuss the structural differences of MCM-22 detemplated by DBD and thermal calcination. The catalytic performance of MCM-22 has been studied using the Fischer–Tropsch (FT) synthesis reaction as a model reaction. The experimental results are well explained based on the porous structure and acidic properties of the MCM-22 zeolites.

## 2. Experimental

### 2.1 Synthesis of the MCM-22 zeolite

MCM-22(P) zeolite with a SiO_2_/Al_2_O_3_ ratio of 50 was synthesized by hydrothermal crystallization according to the reported procedure by Corma *et al.*^8^ HMI (Aldrich) was used as the structure directing agent. Typically, 0.15 g of sodium aluminate (Al_2_O_3_ ≥41%, Sinopharm Chemical Reagent Co., Ltd) and 0.18 g of NaOH (≥96%, Sinopharm Chemical Reagent Co., Ltd) were dissolved in 25.2 mL deionized water. 4.7 g of colloidal silica (Ludox, AS-40, Aldrich) was then slowly added into the solution, and then 1.1 g of HMI (99%, Aldrich) was added dropwise to the gel. The synthesis gel was stirred at room temperature for 1 h. The product was loaded into Teflon lined 50 mL stainless-steel autoclaves and placed in a convection oven at 150 °C. The autoclaves were tumbled at 60 rpm to improve the mixing of the synthesis gel. After 7 days, the autoclaves were quenched in water and the gel was centrifuged and washed with a large quantity of deionized water. The MCM-22(P) was dried at 90 °C overnight. Direct calcination of the sample at 550 °C for 10 h in a muffle furnace under static air resulted in products with a 3D MWW structure. Subsequently, the H-type MCM-22 was obtained by treating Na-type MCM-22 with 1 M NH_4_NO_3_ three times at 80 °C for 2 h followed by calcination at 500 °C for 2 h in a muffle furnace under static air. The H-form MCM-22 is denoted by MCM-22(C).

### 2.2 HMI removal using DBD plasma

The DBD apparatus used in this work was composed of a high voltage generator, electrodes, and a quartz hoop. A high voltage generator (CTP-2000K; Corona Laboratory, Nanjing, China), which can supply a voltage from 0 to 30 kV, was used to generate DBD plasma. The average voltage was 14 kV with a sinusoidal waveform at a frequency of about 22 kHz. The two electrodes were steel plates covered by dielectric-barrier quartz with a thickness of 2.5 mm. The quartz hoop was sandwiched in the middle of the two electrodes. The distance between the two electrodes was about 8 mm. The space between the two plates and the hoop was the discharge gap. Static air was directly applied as the plasma forming gas. A powder sample of MCM-22(P) (0.5 g) was introduced into the DBD reactor. The sample was irradiated with DBD plasma for 4 min per treatment, and was then manually stirred and ground. The sample was treated 20 times. After the exchange of Na^+^ with NH_4_^+^ using the same procedure as in Section 2.1, the NH_4_^+^-type MCM-22 was treated 10 times using DBD, with 4 min per treatment. The H-form MCM-22 treated using DBD is denoted by MCM-22(DBD).

### 2.3 Preparation of the catalysts

The catalysts with a metallic cobalt loading of 10 wt% were prepared by an incipient impregnation method. Co(NO_3_)_2_·6H_2_O (99.0%, Sinopharm Chemical Reagent Co., Ltd) was used as the cobalt precursor. The catalysts were dried at 120 °C for 12 h, and calcined in air at 200 °C for 2 h by increasing the temperature at a controlled heating rate of 2 °C min^−1^.

### 2.4 Characterization techniques

Fourier Transform Infrared spectroscopy (FT-IR) measurements were performed on a PerkinElmer Frontier spectrometer using an attenuated total reflection (ATR) technique. FT-IR spectra were recorded in the range of 2500–4000 cm^−1^ at a wavenumber resolution of 4 cm^−1^. Thermogravimetric analysis was determined on a Q1000DSC + LNCS + FACS Q600SDT thermogravimetric analyzer. The sample was heated in an air atmosphere from room temperature to 800 °C at a ramp rate of 10 °C min^−1^. N_2_ adsorption–desorption isotherms were measured with a Micromeritics ASAP 2020 instrument at −196 °C. Prior to this, 0.1 g samples were outgassed at 300 °C for 12 h. The total surface area was estimated using the Brunauer–Emmett–Teller (BET) method. The external surface area was estimated from a *t*-plot using the adsorption isotherm. The pore size distributions of the micropores were calculated based on the Horvarth–Kawazoe (H–K) method using the data of the adsorption branches. XRD patterns were obtained at room temperature on an X-ray diffractometer (D8 Advance, Bruker) equipped with a Cu Kα radiation source (*λ* = 1.5406 Å) and Ni filter (40 kV, 40 mA). The samples were scanned with a step size of 0.02° and a speed of 0.2 s step^−1^. The crystal size of Co_3_O_4_ over the calcined catalysts was estimated from the Scherrer formula and the (311) diffraction (2*θ* = 36.978°). The crystal size of the metallic cobalt in the reduced catalysts was estimated according to *d*(Co^0^) = 0.75 × *d*(Co_3_O_4_). ^27^Al and ^29^Si MAS NMR experiments were performed on a Bruker AVANCE III 600 spectrometer at a resonance frequency of 156.4 MHz and 119.2 MHz, respectively. ^27^Al MAS NMR spectra were recorded on a 4 mm probe using a small-flip angle technique with a pulse length of 0.5 μs (<π/12), a 1 s recycle delay and a spinning rate of 14 kHz. ^29^Si MAS NMR spectra with high-power proton decoupling were recorded on a 4 mm probe with a spinning rate of 10 kHz, a π/4 pulse length of 2.6 μs, and a recycle delay of 100 s. The chemical shifts of ^27^Al and ^29^Si in MAS were referenced to 1 mol L^−1^ aqueous Al(NO_3_)_3_ and tetramethylsilane (TMS), respectively. The NH_3_-TPD measurements were performed using a Micromeritics Autochem 2920 instrument. Typically, 0.05 g of the sample was first preheated with flowing Ar at 550 °C for 1 h and then cooled to 120 °C. Subsequently, the sample was exposed to an NH_3_–He mixture (5 vol% NH_3_) for 0.5 h. After this, the system was purged for 2 h under a flow of He at the same temperature. Finally, NH_3_-TPD was performed by raising the temperature to 550 °C at a heating rate of 10 °C min^−1^ under a He flow of 30 cm^3^ min^−1^. The reduction degree of the catalyst was estimated using O_2_ titration. 0.1 g of the catalyst was reduced under a flow of high-purity hydrogen (30 cm^3^ min^−1^) at 400 °C for 4 h. After this, the system was purged for 1 h in a flow of Ar at the same temperature. Finally, the sample was reoxidized at 400 °C by pulses of 3% oxygen in argon to determine the reduction degree. On the basis of the oxygen consumed, the reduction degree of the catalyst was estimated by assuming that metallic Co was fully converted to Co_3_O_4_ during the oxygen pulses. H_2_ chemisorption measurements were carried out using a Micromeritics ASAP 2020C. The sample was dried under vacuum at 150 °C for 10 h. The sample was subsequently heated under flowing H_2_ at 400 °C for 4 h, after which the samples were evacuated at that temperature for 30 min. The H_2_ adsorption isotherms were measured at 150 °C. The dispersion of Co was estimated from the total amount of chemisorbed H_2_, assuming a H/Co = 1 atomic ratio stoichiometry. The actual crystal size of Co for the reduced catalysts was calibrated by considering the reduction degree of Co.

### 2.5 FT reaction

The catalytic reaction was tested in a fixed-bed reactor. 0.5 g of catalyst (40–60 mesh diluted with quartz sands) was reduced *in situ* at atmospheric pressure under a flow of pure H_2_ (50 cm^3^ min^−1^) at 400 °C for 4 h. After reduction, the temperature of the catalyst bed was decreased to 190 °C. The syngas (H_2_/CO = 2, 4% Ar as an internal standard) was fed into the reactor and the pressure was increased to 1.0 MPa. The reaction temperature was increased to 235 °C and the reaction conditions were maintained at 235 °C, 1.0 MPa and *W*/*F* = 5.0 g h mol^−1^. The line between the outlet of the reactor and the inlet of the gas chromatograph (GC) was heated at 180 °C to prevent condensation of the products. The hydrocarbons of the effluent products were analyzed using an online GC with an HP-PONA capillary column (0.20 mm × 50 m, 0.5 μm) and a flame ionization detector (FID) (SP-3420A, Beijing Beifen-Ruili Analytical Instrument (Group) Co., Ltd.). The CO, CH_4_, Ar and CO_2_ in the effluent after cooling in an ice-water trap were analyzed using an online GC with a packed activated-carbon column and a TCD detector (SP-3420A, Beijing Beifen-Ruili Analytical Instrument (Group) Co., Ltd.). The hydrocarbon selectivity was calculated on the basis of the carbon number.

## 3. Results and discussion

### 3.1 Effectivity of DBD plasma for template removal from MCM-22

**Fig. 1:**
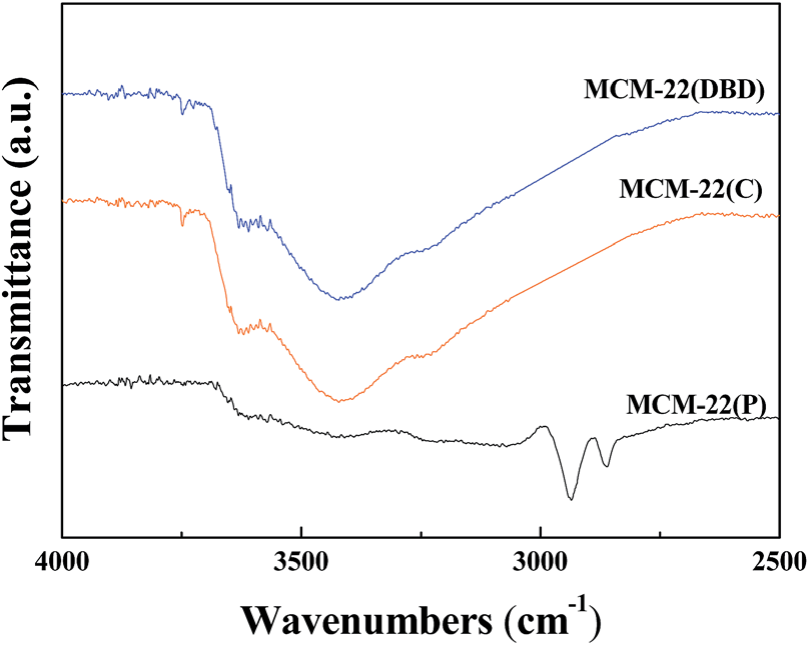
FT-IR spectra of MCM-22(P), MCM-22(C), and MCM-22(DBD).

**Fig. 2:**
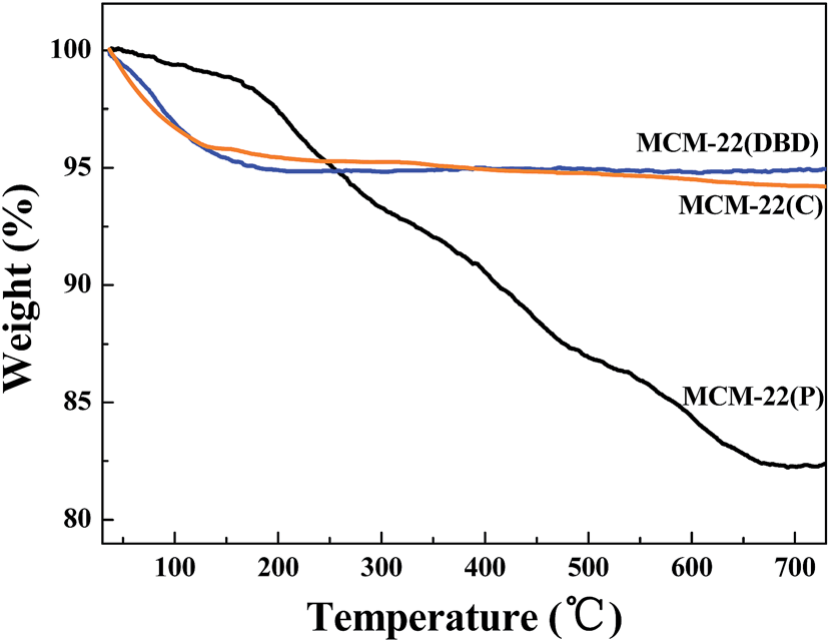
TG curves of MCM-22(P), MCM-22(C), and MCM-22(DBD).

The FTIR spectra of MCM-22(P), MCM-22(C), and MCM-22(DBD) are shown in Fig. 1. The spectrum of MCM-22(P) exhibits two peaks at approximately 2936 cm^−1^ and 2862 cm^−1^, which are assigned to antisymmetric and symmetric stretching vibrations of the C–H bonds in the methylene groups of HMI, respectively.^31^ After DBD treatment or thermal calcination, the C–H stretching vibration disappeared. This result indicated that the HMI in the MWW layers and the interlayer can be effectively removed using DBD treatment. This can be further confirmed by the TG result (Fig. 2). The weight loss from 120 °C to 650 °C for MCM-22(P) is about 16.4%. After template removal, the weight loss of MCM-22 was 1.23% and 1.79% for the sample prepared by DBD treatment and thermal calcination in this temperature range, respectively. Therefore, the effectivity of template removal using DBD plasma is comparable to that of thermal calcination.

### 3.2 Textural and structural properties

**Fig. 3:**
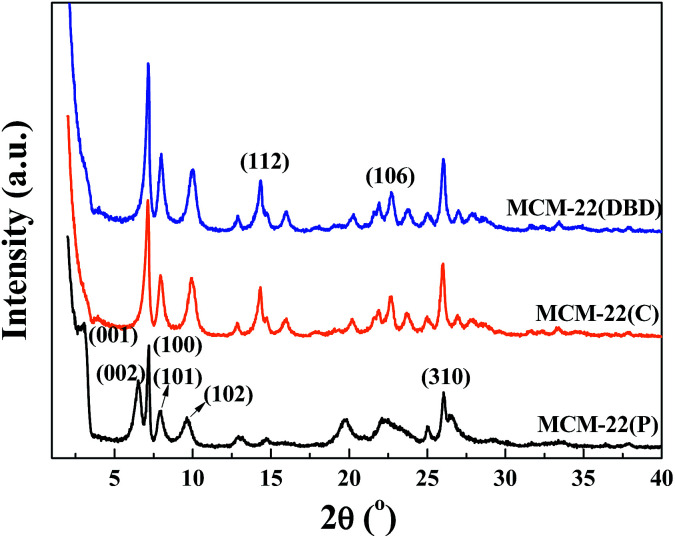
XRD patterns for MCM-22(P), MCM-22(C), and MCM-22(DBD).

The XRD patterns of the MCM-22(P) and MCM-22 samples treated using DBD and thermal calcination are shown in Fig. 3. The XRD pattern for MCM-22(P) is in agreement with those reported in the literature.^8,9^ The (001) and (002) diffraction peaks for MCM-22(P) at 2*θ* values of approximately 3.2 and 6.5° correspond to *d*-spacings of 2.70 and 1.35 nm, respectively. Moreover, two resolved diffraction peaks for (101) and (102) at 2*θ* values of 7.2 and 7.9° indicate the ordered layered structure of MCM-22(P) with the vertically aligned layers being ordered perpendicularly to the *c* axis.^1,32^ Before template removal, the diffraction peaks in the 2*θ* range of 12–30° are broad and some of them overlap. After template removal, both thermal calcination and DBD treatment led to changes in the XRD patterns of the zeolite. The (001) and (002) diffraction peaks had disappeared, and the (002) diffraction peak overlapped with the (100) diffraction of the intralayer. Moreover, several resolved diffraction peaks appeared in the 2*θ* range of 12.8–19.0° (112) and 21.6–23.8° (106), indicating condensation of the terminal silanol groups (Si–OH) on the MWW sheets.^1^ These results indicated that DBD plasma can not only remove HMI, but it is also efficient for inducing condensation of the silanol groups between the adjacent MWW sheets. The intensity of the (100) peak for the DBD treated sample is slightly higher than that of the thermal calcination sample, which indicated that DBD treatment can decrease the collapse of the zeolite. This may be due to the suppression of dealumination due to the lower temperature of the DBD treatment, which can be confirmed by the ^27^Al MAS NMR result.

**Fig. 4:**
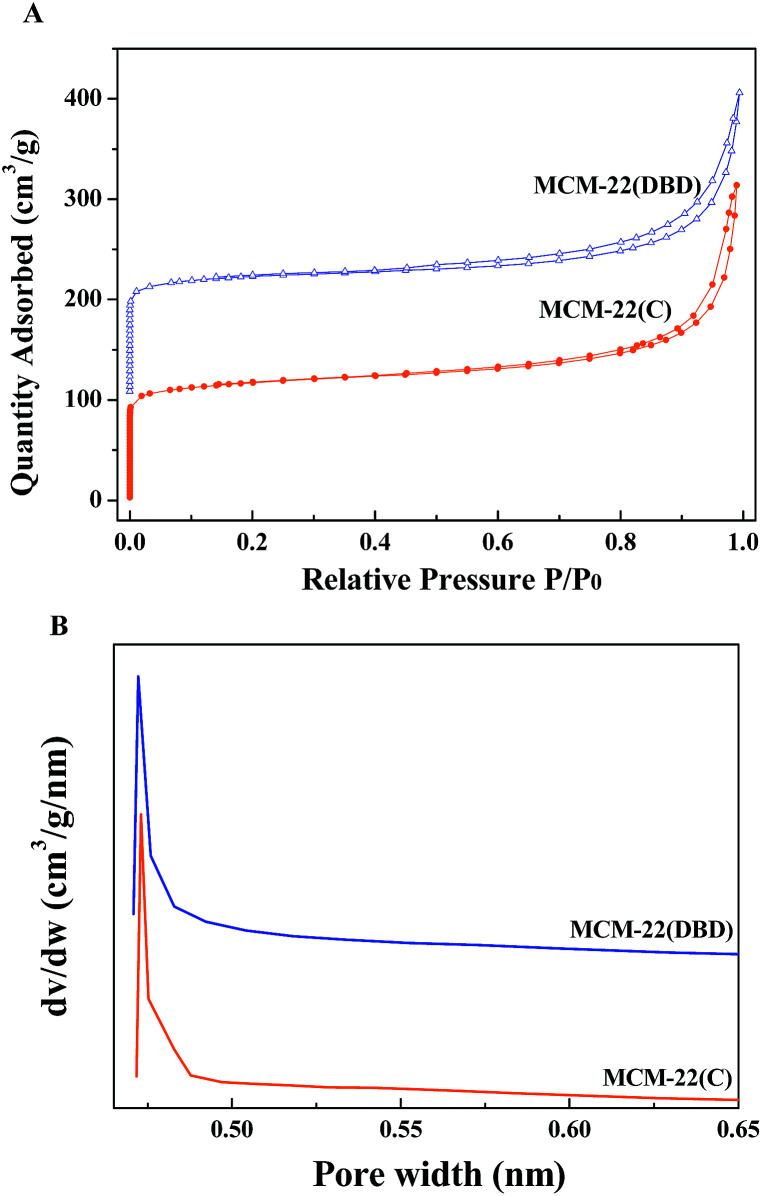
N_2_ adsorption–desorption isotherms (A) and H–K pore size distribution (B) of MCM-22(C) and MCM-22(DBD).

The N_2_ adsorption–desorption isotherms of the thermal calcination and DBD treatment samples are displayed in Fig. 4. Irrespective of the treatment method, MCM-22 shows a type I isotherm, which is typical of microporous materials based on the IUPAC classification.^33^ A peak pore size of about 0.47 nm was confirmed from the narrow pore size distribution calculated by the H–K method. However, the hysteresis loop of MCM-22(DBD) is slightly bigger than that of MCM-22(C), which indicated the presence of mesopores. Table 1 summarizes the surface area and pore volume for the MCM-22 samples treated using DBD and calcination. The total surface area of MCM-22(DBD) and MCM-22(C) is almost the same. However, the external surface area and pore volume of MCM-22(DBD) are larger than that of MCM-22(C). Correspondingly, the micropore area of MCM-22(DBD) is smaller than that of MCM-22(C). The above results may be attributed to the formation of intercrystal mesopores caused by the inhibiting effect of condensation of silanol groups on the external surface of the MCM-22 crystals.^6^

**Table 1:** Porous structure parameters of MCM-22(C) and MCM-22(DBD)

Materials	BET surface area (m^2^ g^−1^)	Micropore area (m^2^ g^−1^)	External area (m^2^ g^−1^)	Pore volume (cm^3^ g^−1^)
MCM-22(C)	396	307	89	0.34
MCM-22(DBD)	403	295	108	0.37

**Fig. 5:**
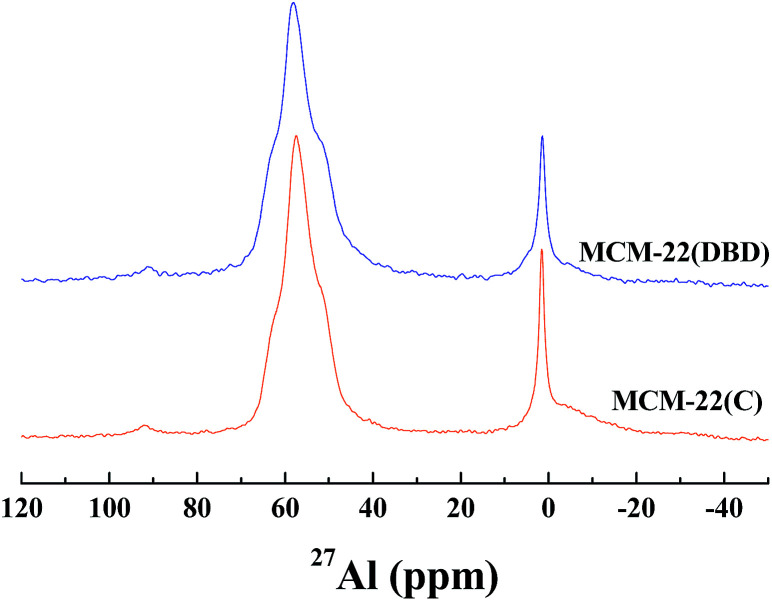
^27^Al MAS NMR spectra of MCM-22(C) and MCM-22(DBD).

**Fig. 6:**
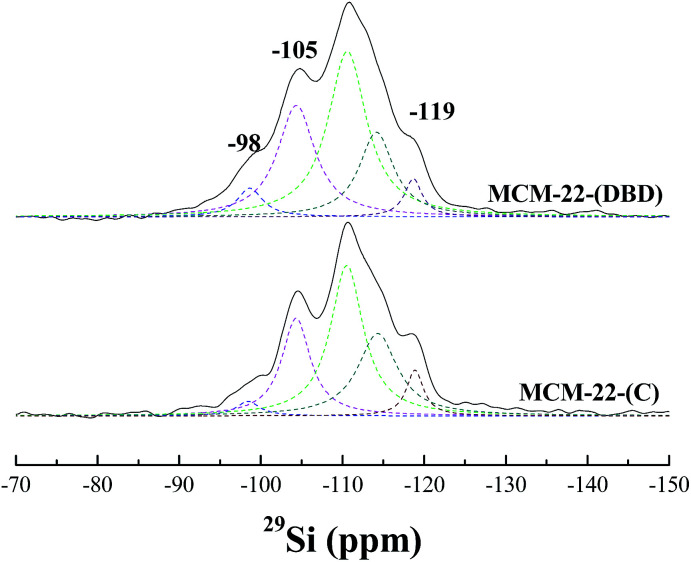
^29^Si MAS NMR spectra of MCM-22(C) and MCM-22(DBD).

The coordination environment of the Al atoms was analyzed using ^27^Al MAS NMR (Fig. 5). Two peaks were present for the calcined and DBD-treated samples. The first peak at about 56 ppm is ascribed to the tetrahedrally-coordinated framework Al species. The second peak at about 0 ppm is ascribed to the octahedrally-coordinated extra-framework Al species.^21,34^ The broad and tailing resonance for MCM-22(C) is assigned to polymeric aluminum species.^35,37^ The amount of extra-framework Al is about 22% after calcination, which is in agreement with previous reports for MCM-22 calcined under dry air.^10,11^ Note that the amount of extra-framework Al is decreased to 17% for the DBD-treated sample, which indicated that DBD-treatment can effectively minimize dealumination of MCM-22. The ^29^Si MAS NMR spectra of the samples are presented in Fig. 6. It is found that the spectrum of MCM-22(C) shows an intense peak at −119 ppm, which is attributed to (Si(OSi)_4_).^34,37^ However, in the case of MCM-22(DBD), a clear decrease in the −119 ppm band is observed. Moreover, the peaks at −98 ppm and −105 ppm from the Si(OSi)_3_OH and Si(0Al) sites located in the vicinity of the surface silanols of MCM-22(DBD) are clearly increased. These results may be attributed to inhibited condensation of the silanol groups on the external surface or in the internal surface of the MWW nanosheets due to the lower temperature process. From the ^27^Al and ^29^Si MAS NMR we can conclude that template removal using DBD treatment can effectively decrease dealumination and inhibit the condensation of silanol groups on the external surface of the MCM-22 crystals.

### 3.3 Acidic properties of MCM-22

**Fig. 7:**
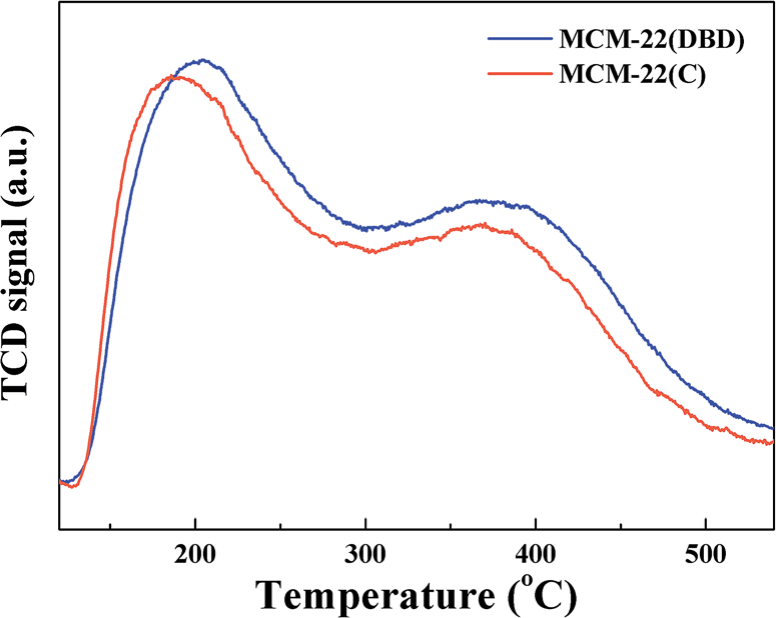
NH_3_-TPD profiles of MCM-22(C) and MCM-22(DBD).

The surface concentration of acidic sites and their strength distribution was estimated using NH_3_-TPD, and the results are shown in Fig. 7. Two clear NH_3_ desorption peaks can be seen for both MCM-22(DBD) and MCM-22(C), which correspond to the weak and strong acidic sites.^9^ The temperature of the first NH_3_-desorption peak for MCM-22(DBD) is about 204 °C, which is clearly higher than that for MCM-22(C) (189 °C). However, the temperature of the second NH_3_-desorption peak for both MCM-22(DBD) and MCM-22(C) is very similar (∼371 °C). These results indicated that the acid strength of the weak acidic sites for MCM-22(DBD) is stronger than that of MCM-22(C), while the acid strength of the strong acidic sites is almost identical for MCM-22(DBD) and MCM-22(C). However, the total number of acidic sites over MCM-22(DBD) is clearly higher than that over MCM-22(C), which can be attributed to the higher concentration of bridging OH groups (Si(OH)Al) for MCM-22(DBD) due to there being more framework aluminium in MCM-22(DBD). Moreover, these results may also be induced by the following effects of extra-framework Al:^35^ (1) compensation of the framework charge of the zeolite resulting in a decrease in the Brønsted acidic site concentration,^38^ (2) hindered access to the effective acid sites by blocking the pores.^36,39^ As mentioned above, template removal from MCM-22 using DBD treatment could decrease the dealumination and effectively preserve the acidic sites.

### 3.4 Particle size and dispersion of cobalt species

From the XRD patterns of the Co-supported catalysts (not shown), the cobalt species over the catalysts were assigned to Co_3_O_4_. Based on the Scherrer formula and the XRD diffractions at 37.0°, the crystal size of Co_3_O_4_ was calculated, and the results are summarized in Table 2. The average crystal size of Co_3_O_4_ over Co/MCM-22(DBD) is slightly larger than that for Co/MCM-22(C). The porous structure parameters in Table 1 show that the proportion of micropores in MCM-22(C) is higher than that in MCM-22(DBD). Moreover, the external surface area of MCM-22(C) is less than that of MCM-22(DBD), which results in the Co species being located at the external surface and intercrystal mesopores of MCM-22(DBD). Thus, Co/MCM-22(DBD) has a larger crystal size of Co_3_O_4_. Furthermore, the above mentioned properties of Co/MCM-22(DBD) result in a higher reduction degree of cobalt over Co/MCM-22(DBD)^40^ (Table 2). Moreover, it has been confirmed that the Co species has a strong interaction with Al_2_O_3_.^41^ Consequently, the large amounts of extra-framework Al_2_O_3_ species will further result in a lower reduction degree of Co over Co/MCM-22(C).

**Table 2:** Crystal size, extent of reduction, and dispersion of cobalt over different catalysts

Co loaded on	XRD		O_2_ titration	H_2_ chemisorption	
	*d*(Co_3_O_4_) (nm)	*d*(Co^0^) (nm)	Reduction degree (%)	Dispersion (%)	*d*(Co^0^)^a^ (nm)
MCM-22(DBD)	23.6	17.7	65	4.3	14.5
MCM-22(C)	22.4	16.8	51	3.3	14.8

a Calculated from *d*(Co^0^) = 96/*D* × reduction degree.

The surface density, dispersion, and mean particle size of metallic Co over the reduced catalysts were also estimated by the H_2_ chemisorption technique, and the results are shown in Table 2. Differing from the technique of XRD, H_2_ chemisorption can measure the density of surface Co^0^ sites over the reduced catalyst. The dispersion of Co over Co/MCM-22(DBD) (4.3%) is higher than that over Co/MCM-22(C) (3.3%). Although the *d*(Co_3_O_4_) calculated from XRD for Co/MCM-22(DBD) is larger than that for Co/MCM-22(C), the reduction degree of Co in Co/MCM-22(DBD) is higher, which results in a higher density of surface Co^0^ sites. Moreover, the actual crystal size of Co for the reduced catalysts was calibrated by considering the reduction degree of Co. The *d*(Co^0^) for Co/MCM-22(DBD) and Co/MCM-22(C) is almost the same (about 14.5 nm).

### 3.5 FT performance

**Fig. 8:**
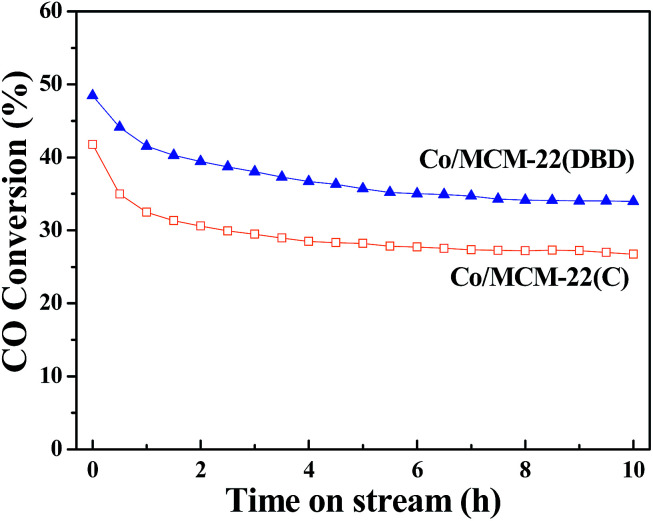
Time-on-stream CO conversion over different catalysts.

The time-on-stream (TOS) catalytic activity results are presented in Fig. 8. CO conversion decreased with the reaction time, and a steady state can be approached at a TOS of about 10 h. Thus, the CO conversion and product distribution after a TOS of 10 h are discussed for the different catalysts. The CO conversion over Co/MCM-22(DBD) is about 34%, which is higher than that over Co/MCM-22(C) (∼27%). This correlates well with the Co dispersion of Co/MCM-22(DBD) (4.3%) and Co/MCM-22(C) (3.3%).

The steady results of the product distribution are summarized in Table 3. The selectivity of CH_4_ and C_2_–C_4_ over Co/MCM-22(DBD) is lower than that over Co/MCM-22(C). It is well-known that higher methane selectivity can be induced by the presence of unreduced cobalt species.^42^ The methane selectivity over the Co/MCM-22 catalysts correlates well with the extent of reduction of the catalysts. Moreover, the actual H_2_/CO ratio over the Co active sites will affect the chain growth probability and then affect the selectivity of hydrocarbons. It is well-known that a higher H_2_/CO ratio in the catalyst pores will be induced due to the lower diffusivity of CO than H_2_ in the micropores.^43^ Therefore, the lower selectivity of C_2_–C_4_ over Co/MCM-22(DBD) could be attributed to the small number of micropores and large number of intercrystal pores, which results in a lower H_2_/CO ratio over the Co active sites in Co/MCM-22(DBD). It should be noted that the selectivity of the C_5_–C_20_ hydrocarbons over Co/MCM-22(DBD) and Co/MCM-22(C) is higher than that over the Co/SiO_2_ catalyst. This result is induced from the cracking of long-chain FT hydrocarbons over the acidic sites of MCM-22 zeolite.^32,44^ Due to the higher concentration and stronger acidic sites over Co/MCM-22(DBD), the selectivity of C_5_–C_20_ hydrocarbons over Co/MCM-22(DBD) is higher than that over Co/MCM-22(C). The slightly high selectivity of C_21_+ hydrocarbons can be reasonably attributed to higher CO conversion, which results in the high space velocity of long-chain FT hydrocarbons over the acidic sites of MCM-22(DBD).

**Table 3:** Main results of FT synthesis over different catalysts^a^

Catalysts	CO conversion (%)	Hydrocarbon distribution (%)			
		C_1_	C_2_–C_4_	C_5_–C_20_	C_21_+
Co/MCM-22(C)	26.6	15.4	13.3	66.6	4.7
Co/MCM-22(DBD)	34.0	14.8	9.4	70.5	5.2

a Operating conditions: *W*/*F* = 5.02 g h mol^−1^, *P* = 1.0 MPa, *T* = 235 °C, TOS = 10 h.

## 4. Conclusions

In summary, the template of HMI in two-dimensional layered MCM-22 zeolite can be effectively removed through DBD plasma treatment. Compared with calcination, DBD treatment could preserve the structure well and decrease the formation of extra-framework aluminium due to the lower temperature process, which results in a higher concentration of acidic sites over MCM-22(DBD) than over MCM-22(C). Moreover, condensation of the silanol groups on the external surface of the MCM-22 crystals can be inhibited, which decreases the formation of agglomerated crystals and increases the number of intercrystal pores. Consequently, Co/MCM-22(DBD) shows a higher CO conversion and C_5_–C_20_ hydrocarbon selectivity in the FT synthesis reaction.

## Conflicts of interest

There are no conflicts to declare.

## Supplementary Material
